# Distribution of Serological Response to *Burkholderia pseudomallei* in Swine from Three Provinces of Vietnam

**DOI:** 10.3390/ijerph17145203

**Published:** 2020-07-18

**Authors:** Michael H. Norris, Hang Thi Thu Tran, Morgan A. Walker, Andrew P. Bluhm, Diansy Zincke, Trinh Thanh Trung, Nga Vu Thi, Ngoc Pham Thi, Herbert P. Schweizer, Fred Unger, Jason K. Blackburn, Nguyen Thi Thu Hang

**Affiliations:** 1 Spatial Epidemiology and Ecology Research Lab, Department of Geography, University of Florida, Gainesville, FL 32611, USA; m.walker@ufl.edu (M.A.W.); abluhm@ufl.edu (A.P.B.); dzincke@ufl.edu (D.Z.); jkblackburn@ufl.edu (J.K.B.); 2 Emerging Pathogens Institute, University of Florida, Gainesville, FL 32611, USA; hschweizer@ufl.edu; 3 National Institute of Veterinary Research, Hanoi 10000, Vietnam; thuhang1207@gmail.com (H.T.T.T.); ngacvd@gmail.com (N.V.T.); minhngoc27169@gmail.com (N.P.T.); hangchau71@gmail.com (N.T.T.H.); 4 VNU-Institute of Microbiology and Biotechnology, Vietnam National University, Hanoi 10000, Vietnam; tttrung@vnu.edu.vn; 5 Department of Molecular Genetics and Microbiology, University of Florida, Gainesville, FL 32611, USA; 6 International Livestock Research Institute, Hanoi 10000, Vietnam; F.Unger@cgiar.org

**Keywords:** melioidosis, Whitmore, swine, infectious disease, zoonosis, Vietnam, outbreak, *Burkholderia pseudomallei*, interdisciplinary

## Abstract

(1) Background: *Burkholderia pseudomallei* is an environmentally mediated saprophytic pathogen that can cause severe disease in humans. It is well known that *B. pseudomallei* survives in tropical moist soil environments worldwide, but melioidosis is gaining recognition as a public and veterinary health issue in Vietnam. The contribution of animals to human disease is unknown, necessitating further investigation. (2) Methods: Swine sera were collected from two populations, one grazing and one commercially farmed, from three provinces in Vietnam. ELISAs utilizing *B. pseudomallei* capsular polysaccharide (CPS), outer polysaccharide (OPS), and Hcp1 protein were used to screen serum samples. Positive samples were mapped to the commune level. Seroprevalence calculations and pig population estimates were used to approximate number of swine exposures per commune. (3) Results: Grazing pigs from Hoa Binh had significantly higher seropositivity levels (11.4%, 95% CI: 9.7–13.1) compared to farmed pigs from Ha Tinh and Nghe An (4%, 95% CI: 3.3–4.7). Average swine seropositivity rates were ~6.3% (95% CI: 5–7.9), higher than previously identified in Vietnam (~0.88%). (4) Conclusions: Initial serological sampling identified a significant number of seropositive and potential melioidosis infections occurring in swine in Vietnam. This work is a critical step in understanding the role swine may play in the epidemiology of human melioidosis in Vietnam.

## 1. Introduction

*Burkholderia pseudomallei* is a dangerous Gram-negative bacterium that causes melioidosis [1,2]. Melioidosis is a neglected tropical disease that has been well-studied in Thailand and Australia but is believed to be endemic in tropical regions globally, including Vietnam [3,4,5]. Although the organism can cause serious diseases in humans and animal models, predisposing conditions such as diabetes and alcoholism are usually prerequisite for acute and chronic disease in humans [6,7]. Therefore, the notion that *B. pseudomallei* is an environmental saprophyte that accidentally infects humans has taken root. The organism, however, has a unique cache of virulence factors that allow intracellular replication in the cytoplasm of many cell types [8] and production of acute lethal infections in numerous animal models [9,10,11,12]. *Burkholderia pseudomallei* survives well in soils throughout the tropics and can be isolated at high concentrations from soil [13]. Heavy rainfall events such as monsoon rains or tropical cyclones often precede a rise in observed human cases [14,15,16]. The link between rainfall events and human cases suggests persons exposed to contaminated waters are infected through breaks in the skin that result in systemic infections. Human aerosol exposure has not been entirely proven and demonstration of naturally aerosolized *B. pseudomallei* has been limited [17].

The role of water as a means of pathogen dispersal and mechanism of human disease is widely accepted, and the role of human activities in global pathogen dispersal has been postulated. Molecular clock studies have demonstrated the transfer of *B. pseudomallei* to Southeast Asia from Australia, from Southeast Asia to Africa, and then a coincidence of timing in movement of *B. pseudomallei* to the Americas during the height of the slave trade [18]. Melioidosis as a zoonosis has not been well studied, and the animal role in epidemiology of melioidosis takes a backseat to environmentally mediated mechanisms. A glaring ignorance to the role zoonoses can play in *B. pseudomallei* adaptation and lifestyle is evident in the clonal expansion of the glanders-causing bacterium *Burkholderia mallei* [19,20]. *Burkholderia mallei* is a pathogenic host-adapted descendent of *B. pseudomallei* that no longer survives in the environment because of within-host gene loss and genome decay. The implication is animals likely play an important role in exposure and carriage of *B. pseudomallei*. By way of multiple and continuous exposures, the pathogen can be dispersed over larger distances and begin evolving within infected animals. It can be hypothesized that animal exposure to *B. pseudomallei* in the environment is happening at increased levels compared to humans. Additionally, an important component of one health initiative is the contribution of animal health to human health. A report of wild caught rats in Sri Linka found serological evidence of exposure to environmental *B. pseudomallei* [21]. Likewise, non-human primates in Indonesia were reported to have serological evidence of exposure to *B. pseudomallei* [22]. For these reasons it has become important to study natural animal melioidosis exposures as true indicators of environmental exposure levels to better understand human risk and disease prevalence.

Swine are an important protein source in South East Asia. Vietnam is no exception with ~22 million pigs valued at nearly 10% of the agricultural sector and swine acting as a major income source for Vietnamese farmers [23]. Evidence of exposure in swine can be presumed recent because these animals lead short lives before going to market (approximately 6 months). A random swine tracheal swabbing study showed *B. pseudomallei* isolation from ~0.88% of the swine tested [24]. The true percentage of infected swine is likely higher due to the propensity of false negatives using direct culture methods. Even at ~0.88%, ~194,000 swine in the countrywide at any given time would have easily isolatable *B. pseudomallei* present in the trachea. In the absence of animal health controls or in a less organized operation, infected animals could be slaughtered and sent to market. It is currently unknown whether melioidosis is acquired by meat processors, meat market workers, or consumers during handling or consumption of infected animals as they enter the food supply.

Here, we screened swine serum samples for antibodies reactive to various *B. pseudomallei* antigens with well characterized utility in measuring melioidosis exposure. Our objectives were to (1) estimate seroprevalence in sampled grazing and commercial swine populations from three provinces in Vietnam; and (2) estimate the total number of swine likely exposed across these provinces using swine population estimates.

## 2. Methods

### 2.1. Isolation and Purification of Polysaccharide ELISA Antigens

For this study, three antigens were used to investigate swine exposure to *B. pseudomallei*: capsular polysaccharide (CPS) [25,26], type A outer polysaccharide (OPS; derived from lipopolysaccharide LPS) [12,27,28,29], and recombinant hemolysin coregulated protein 1 (Hcp1) [30,31,32]. Antigens were purified at the University of Florida Emerging Pathogens Institute from select-agent exempt Bp82 mutants. CPS was isolated from Bp82 Δ*wbiI-E*, and OPS was isolated from the Bp82 Δ*wcb* mutant using a protocol adapted from Lam et al. [33] and as previously described [29]. Briefly, 200 μL of starter cultures grown overnight in LB at 37 °C were spread on TSA plates and allowed to grow for 72–96 h at 30 °C. The resultant bacterial lawns were harvested in PBS, heat-treated at 110 °C for 15 min, and then mixed to 50% phenol. The lysate mixture was stirred at 60 °C for 1 h and then dialyzed against water with multiple water changes at 4 °C until the odor of phenol dissipated. The dialyzed lysate was treated with DNase, RNase, and proteinase K and then lyophilized to dryness. The LPS sample was further treated to remove the OPS from the lipid portion of the LPS molecule. It was hydrolyzed with 1 M acetic acid for 1 h at 100 °C and then lyophilized again. Then, 100% ethanol was added, and the precipitated lipid A pelleted out of the solution by repeated rounds of centrifugation and washing. The resulting OPS material was resuspended in ammonium acetate and purified by size exclusion chromatography using a HiPrep Sephacryl S-300 HR attached to an Agilent refractive index detector. The A_280_ absorbing material eluting from the column headspace was the hydrophobic cleaved lipid A fragment, while a prominent peak only detectable by refractive index was the cleaved OPS. This material was lyophilized, and dry mass was determined. CPS was also column purified, lyophilized, and mass determined.

### 2.2. Production of Recombinant Hcp1 Protein Antigen

Hcp1 from 1026 bg DNA was amplified by PCR, cloned into pET21b, and then expressed in *E. coli* expression strain BL-21 (DE3). Hcp1 protein was purified using cobalt exchange chromatography. The purified protein was visualized by silver stain and passed through a Pierce high capacity endotoxin removal column (Thermo Fisher, Waltham, WA, USA) to remove any contaminating *E. coli* endotoxin. The Pierce BCA protein assay kit (Thermo Fisher, Waltham, WA, USA) was used to determine the concentration of purified protein.

### 2.3. Preparation of ELISA Plates

Flat bottom Immulon4 HBX plates were coated with 2 μg/mL OPS, 2 μg/mL CPS, or 4 μg/mL purified Hcp1 in PBS overnight at room temperature at the Emerging Pathogens Institute. The following day the plates were washed three times with PBS and allowed to dry. The plates were sealed with parafilm and frozen at −80 °C until shipped to Hanoi, Vietnam. Plates were shipped on ice where they were stored at 4 °C until used.

### 2.4. Serum Sample Acquisition and Study Locations

Using G*Power [34], the number of total samples needed to reject the null hypothesis with 80% power and to detect a small effect size difference between unexposed and exposed swine populations with *p* < 0.05 was calculated as 1116 samples. The allocation ratio was estimated as 0.2 for exposed/swine based on the observed childhood seropositivity rates of 11–24% measured in melioidosis endemic provinces of Thailand [35]. Ha Tinh and Hoa Binh provinces were identified as areas of increased human melioidosis incidence in Vietnam. These two provinces and an additional province, Nghe An province, for a total of three provinces, were sampled in this study. Swine were defined as grazing, belonging to small farms, or farmed, belonging to large commercial farming operations. Grazing swine (*n* = 352) from 6 communes in Hoa Binh Province were sampled in June 2018. Farmed swine (*n* = 773) from 7 districts in Ha Tinh Province (*n* = 743) and 1 in Nghe An Province (*n* = 30) were sampled from August to October 2019 for a total of 1125 samples. Samples were transported on ice from the study areas to the National Institute for Veterinary Research (NIVR) in Hanoi, Vietnam and frozen until assayed at the end of October 2019. Sample location (commune, district, province), location code, sample date, and numbers of total samples are found in Table 1.

### 2.5. Swine Serum ELISA

Blocking was done for 1 h using BLOTTO blocking grade non-fat skim milk powder (Santa Cruz Biotechnology, Dallas, TX, USA) at 5% (*w*/*v*) in 1X PBST (PBS + 0.005% Tween 20; Pierce 20X PBS Tween-20 Buffer). Serum samples were diluted 1:500 in blocking solution and 200 μL was incubated for 1 h at room temperature. Plates were washed three times with blocking solution and 100 μL rabbit anti-swine IgG-peroxidase conjugated detection antibody (Sigma Aldrich, St, Louis, MO, USA; SAB3700427) was added at 1:15,000 in blocking buffer for 1 h. Following three washes with PBST, 100 μL of substrate (Pierce 1-Step Ultra TMB ELISA solution) was added and developed for 30 min, and then 100 μL 1N HCl was added to stop the reaction. Plates were read at A_450_ within 30 min of adding stop solution. For CPS and OPS antigen ELISAs, samples were run in duplicate on two separate plates and the values averaged. Hcp1 antigen ELISAs were run in triplicate on 3 different plates.

### 2.6. Statistical Analysis of ELISA Data

Each antigen was analyzed separately. One absorbance value was calculated for each animal by taking the average measurement from serum samples run in duplicate using either the anti-*B. pseudomallei* CPS or the OPS, or in triplicate with the Hcp1 ELISA. Because the random observed swine tracheal isolation rate of live *B. pseudomallei* is low (0.88% [24]) it was assumed that the majority of the population would not have antibodies toward purified *B. pseudomallei* antigens and that if positive, the absorbance values would manifest in a binomial population distribution. Although not ideal, this methodology is acknowledged by the World Organization for Animal Health (formerly the OIE) as a legitimate means to establish cutoff when known positive samples may not be available or if the diagnostic assay is not validated, as in our prototype ELISAs measuring swine exposure to *B. pseudomallei* [36]. Thus, the absorbance values from seronegative population animals would be defined by sample mean (μ) and sample standard deviation (σ) of the population’s Gaussian distribution [37]. An absorbance threshold (λpos) was set at three sample standard deviations from the mean (μ + 3σ) of the suspected seronegative population to conservatively exclude ≥99.7% of seronegative members from being classified as seropositive using Stata version 13 (StataCorp LP., College Station, TX, USA). The animals having A_450_ measurements outside this threshold in any of the three purified antigen ELISAs were considered seropositive.

### 2.7. Mapping and Estimation of Seroprevalence and Case Numbers

Local seroprevalence of melioidosis-positive swine was mapped to the commune (sub-district unit) using QGIS version 3.4 (https://qgis.org/en/site/). Political boundaries were downloaded from GADM (http://www.gadm.org). Seroprevalence was first determined for test populations per spatial unit. Next, we calculated crude expected case numbers in swine in the sample units using estimates of pig populations from the Gridded Livestock of the World (GLW) dataset [38] and multiplying by the approximate seroprevalence determined for the sample populations. Total swine from GLW were summated to the commune using the zonal statistics routine in QGIS. Comparison of grazing and farmed pigs was done by first conducting Shapiro–Wilk’s test for normality and then a Mann–Whitney U Test to compare the raw seroprevalence from “Graze” and “Farm” samples with α = 0.05. Exact binomial confidence intervals were calculated using the epitools package in R for all samples by ELISA test type and by commune.

### 2.8. Ethics Statement

All work in this manuscript took place in Vietnam following Article 21 under the Vietnam National Law governing animal welfare. The National Institute for Veterinary Research is approved for animal sampling and was conducted as per the guidance of the Ministry of Agriculture and Rural Development Circular 7. Swine serum sampling and interaction with livestock and livestock owners were done in accordance with Circular 7 and Article 21.

## 3. Results

### 3.1. CPS and OPS ELISAs

**Figure 1:**
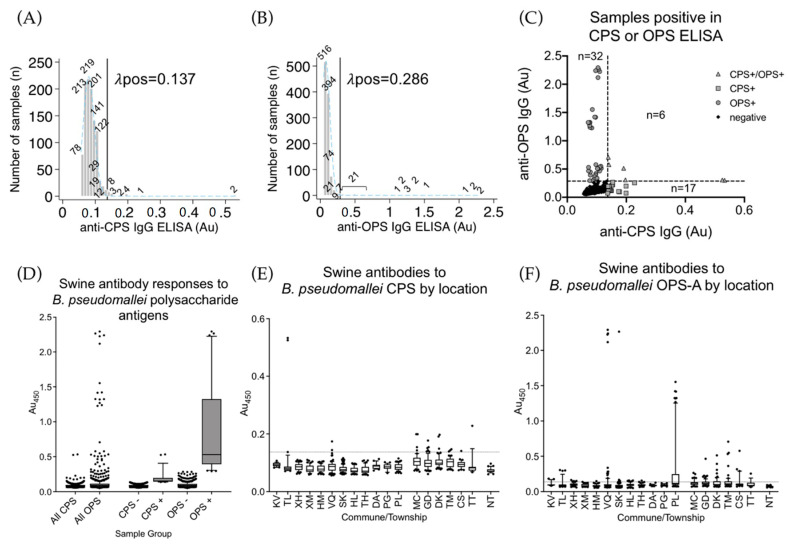
Anti-CPS and OPS IgG ELISAs. (**A**) A histogram of 1056 swine serum samples from Vietnam tested for anti-CPS IgG antibodies. The cutoff threshold for the CPS ELISA assay is indicated by the vertical black line at 0.137 Au. The light blue dashed line indicates the normal distribution of the CPS seronegative population. The numbers above each bar indicate the total number of samples in each of 53 bins. (**B**) A histogram of 1056 swine serum samples tested for anti-OPS IgG antibodies. The cutoff threshold for the OPS ELISA assay is indicated by the vertical black line at 0.286 Au. The light blue dashed line indicates the normal distribution of the CPS seronegative population. The numbers above each bar indicate the total number of samples in each of 53 bins. (**C**) A comparison of anti-CPS IgG and anti-OPS IgG for the 1056 serum samples. Grey triangles are samples that were positive for CPS and OPS assays. Grey squares are samples positive in only the CPS assay, grey circles are only positive in OPS assay, and the black dots are the seronegative population. (**D**) The absorbance values obtained in the CPS and OPS-A ELISAs of all samples (all), samples not passing cutoff (CPS- and OPS-), and those passing cutoff (CPS+ and OPS+). (**E**) Swine antibody responses by commune/township as detected in the CPS ELISA. The dotted line indicates calculated cutoff value of 0.137 Au. (**F**) Swine antibody responses by commune/township as detected in the OPS-A ELISA. The dotted line indicates the calculated cutoff value of 0.286 Au. Boxes are the interquartile range. Whiskers indicate the 10th and 90th percentiles. Black dots indicate individual samples outside of these ranges. Location codes can be found in Table 1.

Table 2 contains a summary of results from the ELISAs run in this study. The CPS ELISA was run on 1056/1125 samples in duplicate (Figure 1A). A total of 23/1056 (2.17%, 95% CI: 1.4–3.3) passed the cutoff of 0.137 Au. Two samples were more than double the next highest samples. These two samples were from Thuong Loc, Ha Tinh and were sampled on 18 September 2019 (Table 1). Of the samples passing the cutoff in the CPS ELISA, 5/23 were from Ha Tinh and the remaining 18/23 samples were from Hoa Binh (Figure 1E). CPS is a T-independent antigen, and antibody responses to these antigens can be variable [37]. The OPS was run in tandem with the CPS ELISA and in duplicate with the same 1056/1125 samples (Figure 1B,D). A total of 38/1056 (3.60%, 95% CI: 2.6–4.9) samples passed the cutoff of 0.286 Au. Absorbance of the OPS seropositive population was much higher than the CPS population; however, the absorbance values of the seronegative population were still very low. A total of 11/38 passing cutoff were from Hoa Binh Province, and 27/38 were from Ha Tinh Province (Figure 1F); 61/1056 (5.78%, 95% CI: 4.4–7.4) were positive in either the CPS or OPS ELISA, and six of the samples passed cutoff in both the CPS and OPS assays (Figure 1C). Of the six samples passing cutoff in both assays, four were from Hoa Binh (one from Tan Minh, one from Cao Son, and two from Doan Ket) and two were from Thuong Loc, Ha Tinh (the same two samples with the highest response in the CPS ELISA). Four subsets within the OPS test were visible: very high positives, high positives, mid positives, and the seronegative population. This was somewhat visible in the CPS assay, but the low signal levels prevented a clear picture of this phenomenon. OPS is normally attached to the highly immunogenic lipid A and so the host antibody response to OPS sugars was more consistent and stronger.

### 3.2. Hcp1 ELISA

**Figure 2:**
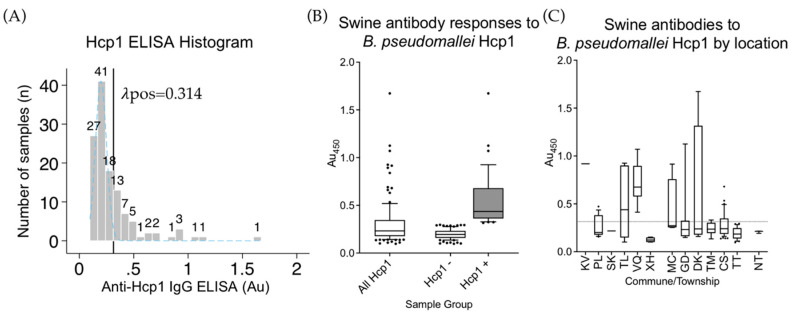
Anti-Hcp1 IgG ELISA. (**A**) A histogram of 133 swine serum samples from Vietnam tested for anti-Hcp1 IgG antibodies. The cutoff threshold for the Hcp1 ELISA assay is indicated by the vertical black line at 0.314 Au. The light blue dashed line indicates the normal distribution of the CPS seronegative population. The numbers above each bar indicate the total number of samples in each of 22 bins. (**B**) The absorbance values obtained from the Hcp1 ELISA of 133 swine serum samples (all Hcp1), those not passing cutoff (Hcp1-), and those passing cutoff (Hcp+). (**C**) Swine antibody responses by commune/township as detected in the Hcp1 ELISA. The dotted line indicates calculated cutoff value of 0.314 Au. Boxes are the interquartile range. Whiskers indicate the 10th and 90th percentiles. Black dots indicate individual samples outside of these ranges. Location codes can be found in Table 1.

The Hcp1 ELISA was run on 68 samples from Hoa Binh not tested by CPS or OPS due to reagent limitations, and also the 55 samples positive in either CPS or OPS ELISAs. Using the same methods as above, a cutoff for the swine anti-Hcp1 IgG ELISA at 0.314 Au was determined (Figure 2A); 39/94 (41.5%, 95% CI: 31.4–52.1) of the Hcp1 tested samples were considered positive by this test but it is important to remember CPS or OPS positive samples were also retested. If only previously untested samples from Hoa Binh were analyzed, 16/68 (23.5%, 95% CI: 14.1–35.4) were found positive (Figure 1B,C).

**Figure 3:**
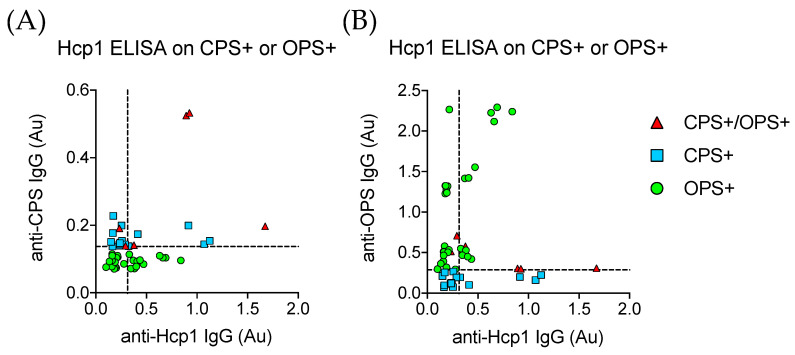
Hcp1 ELISA on Hoa Binh and CPS+/OPS+ Samples. (**A**) The anti-CPS IgG ELISA absorbance values of all samples passing cutoff in either the CPS or OPS ELISA compared to the measured absorbance in the anti-Hcp1 IgG ELSA. Dashed lines on each axis represent the cutoff values for their respective assays. (**B**) The anti-OPS IgG ELISA absorbance values of all samples passing cutoff in either the CPS or OPS ELISA compared to the measured absorbance in the anti-Hcp1 IgG ELSA. Dashed lines on each axis represent the cutoff values for their respective assays. Red triangles are samples that were positive for CPS and OPS assays. Light blue squares are samples positive in only the CPS assay, and green circles are only positive in the OPS assay.

Samples passing cutoff in the CPS or OPS ELISAs were retested using Hcp1 as ELISA antigen. There was poor correlation between CPS+ sample absorbance and Hcp1 ELISA absorbance levels (Figure 3A); 4/6 (66.7%, 95% CI: 22.3–95.7) of the double CPS+/OPS+ serum samples positive by Hcp1 ELISA and 0/17 (0.0%, 95% CI: 0–19.5) of the CPS+ samples were reactive in the Hcp1 assay. A total of 17/31 (54.8%, 95% CI: 36.0–72.7) of the OPS+ samples were positive by the Hcp1 test. Correlation between the OPS and Hcp1 assay was better than the CPS and Hcp1 assay but still poor, indicating antibody responses to Hcp1 were variable but more reliable than antibody responses to CPS. Our data agreed with previous findings that an assay incorporating both OPS and Hcp1 would be more sensitive and specific in detecting antibody responses to *B. pseudomallei* infection [30,32].

### 3.3. Seroprevalence and Estimated Cases in Ha Tinh and Nghe An Provinces

**Figure 4:**
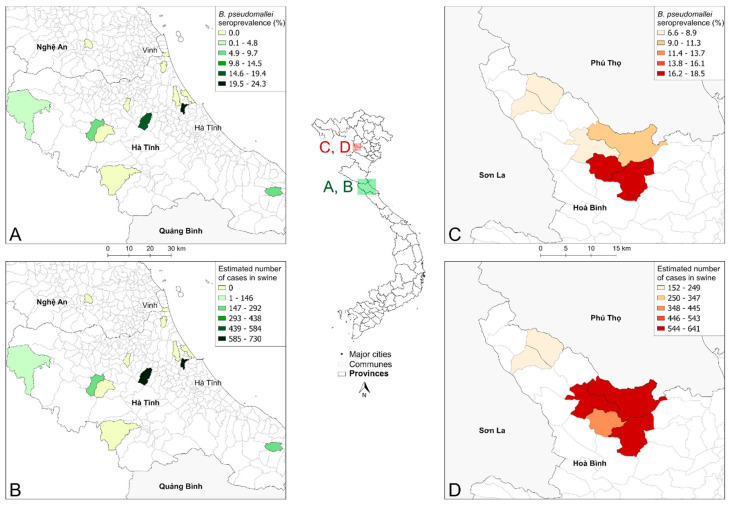
Estimated *B. pseudomallei* swine exposures in Ha Tinh, Nghe An, and Hoa Binh Provinces, Vietnam. (**A**) The seroprevalence of melioidosis in swine mapped to the communes of Ha Tinh and Nghe An Provinces. (**B**) The estimated number of total seropositive animals in the sampled communes in Ha Tinh and Nghe An. (**C**) Seroprevalence of melioidosis in swine in the six communes tested in Hoa Binh Province. (**D**) The estimated number of total seropositive animals in the sampled communes in Hoa Binh Province. Inset map identifies the locations of panels A–D within Vietnam.

Ha Tinh and Nghe An are provinces in the North Central Coast region of Vietnam (Figure 4, inset map). The animals sampled in these provinces were all farmed swine. At the commune level, the estimated seroprevalence was highest in Phu Luu, Loc Ha where 18/74 samples were positive (24.3%, 95% CI: 15.1–35.7); 5/32 (15.6%, 95% CI: 5.3–32.8) were positive in Thuong Loc, Can Loc and 6/113 (5.3%, 95% CI: 2.0–11.2) in Vu Quang (Figure 4A). Other communes tested ranged from 0.00% to 5.0% seroprevalence. Using GLW data, Phu Luu was predicted to have approximately 730 seropositive swine (Figure 4B). Thuong Loc was predicted to have 656, Ky Van 228, and Vu Quang 149 seropositive swine. The remaining communes sampled had between 0 and 110 seropositive swine. In the sampled communes, the total number of seropositive swine were estimated to be approximately 2000 animals.

### 3.4. Estimated Swine Expposures to Environmental B. pseudomallei in Hoa Binh Province

Hoa Binh province is in the mountainous Northwest region of Vietnam and is ~285 km from the Ha Tinh sample site (Figure 4, inset map). Samples available from Hoa Binh Province were limited to communes in Da Bac district in the northwest of the province. The animals sampled during this trip were all grazing swine. Seroprevalence was highest in Trung Thanh, where 7/38 (18.4%, 95% CI: 7.7–34.3) samples were positive and in Cao Son, where 13/76 (17.1%, 95% CI: 9.4–27.5) samples were positive. Seroprevalence ranged from 4/60 (6.7%, 95% CI: 1.8–16.2) to 6/60 (10.0%, 95% CI: 3.8–20.5) in Doan Ket, Giap Dat, Muong Chieng, and Tan Minh (Figure 4C). Cao Son, Doan Ket, and Tan Minh were each estimated to have 544–641 seropositive animals based on GLW analysis (Figure 4D). Trung Thanh, Giap Dat, and Muong Chieng were estimated to have 414, 238, and 152 seropositive animals, respectively. The estimated number of seropositive animals in the six sampled communes in Da Bac District was approximately 2500 animals.

### 3.5. Grazing Versus Farmed Swine

**Figure 5:**
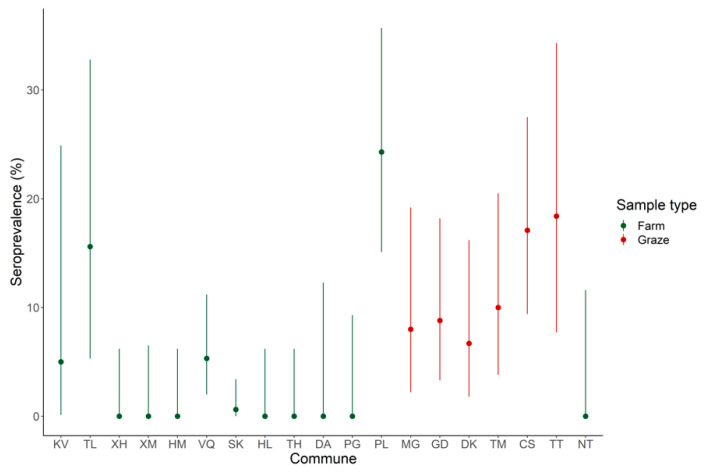
Seroprevalence in grazed versus farmed swine sampled in Vietnam analyzed in this study was significantly different. Seroprevalence rates of grazed (red) and farmed (green) are shown by individual commune with the 95% confidence intervals. Order of communes matches Figure 1.

Many swine in Vietnam can graze freely but farmed swine are becoming much more common. The samples from Ha Tinh and Nghe An were from farmed pigs and samples from Hoa Binh were from free grazing pigs. The dichotomy of samples allowed comparison of seroprevalence rates in the two populations. Seropositivity for farmed and grazed animals was not normally distributed (Shapiro–Wilk Test, *p* = 0.002 and W = 0.817). Farmed and grazed populations were significantly different from one another based on the Mann–Whitney U Test (*p* = 0.0094 and W = 10); grazed animals exhibited a significantly higher seroprevalence rate than farmed animals, although there was notable variation in the farmed samples when evaluated by commune (Figure 5).

## 4. Discussion

This study used three different ELISAs to successfully measure swine seropositivity to specific *B. pseudomallei* antigens in 1125 serum samples from three provinces in Vietnam. The OPS ELISA gave the strongest signal with the least background, followed by the Hcp1 ELISA. The CPS ELISA found seropositive samples, but the readings were lower and limited. Discussed above, CPS is a polysaccharide. Polysaccharides (CPS and LPS) are poor immunogens and predominantly activate antibody secretion without involving T-cells. CPS would be considered a T-cell independent type II antigen (TI-2). Memory cells are not produced to TI-2 antigens. On the other hand, LPS (from which the OPS is derived) strongly activates B cells to produce antibodies and through its innate immunogenicity generates memory cells. The nature of the antigen explains why we see more seropositive animals in the OPS ELISA compared to the CPS. The protein antigen Hcp1 ELISA combined with OPS in separate ELISAs as done here, or in a combined assay, are complimentary in their detection of exposure to melioidosis. Interestingly, samples seropositive in the OPS ELISA formed three clusters of anti-OPS IgG. This could indicate different levels of exposure or different times since exposure. It could indicate the difference between exposure and true infections; however, more intensive sampling is required to answer this question.

We found 71/1125 (6.3%, CI: 5.0–7.9) randomly sampled swine in three provinces were considered seropositive. We found seropositive swine in multiple communes across the area sampled, with seropositivity rates as high as 18/74 (24.3%, 95% CI: 15.1–35.7) among farmed swine in Phu Luu and 7/38 (18.4%, 95% CI: 7.7–34.3) in grazing swine in Trung Thanh. Our data also show that many of the seropositive samples from Ha Tinh were clustered in the later sampling dates between 9–12 October 2019. This season in Vietnam is associated with heavy rainfall. Mid-October 2019 Ha Tinh was beset by heavy flooding, a time coinciding with the end of our sampling window. Notably, our samples were collected in June 2018 and Summer/Fall 2019, while the ~0.88% PCR-based prevalence were from pigs collected from August to October in 2016 and 2017, indicating *B. pseudomallei* detection in swine across four different years. Pigs grow rapidly and have mature immune systems 56 days after birthing [39,40]. The ages of the animals sampled for this study were unknown, but in Vietnam and elsewhere, pigs are market-ready by 6 months of age if not before. The rapid growth and quick time to market provide a short window for bacterial exposure/infection and antibody development. To observe such levels of seroprevalence in a random sampling of pigs at market age or younger indicates the potential for a high level of environmental exposure. There are 9111 communes across 58 provinces in Vietnam (as of 2020). The number of animal exposure events were estimated for each commune sampled in this study based on our crude seroprevalence rates. In the 19/9111 communes in 3/58 provinces of Vietnam sampled in this study an estimated 3850–5830 animals are receiving a high enough exposure to *B. pseudomallei* to result in measurable antibody production. These estimates are preliminary, given an unknown sensitivity of the prototype swine ELISA assays used here, but indicate that future work should investigate swine as sentinels for human risk. Sensitivity of human melioidosis ELISAs targeting Hcp1 and OPS have been determined as 83% and 71.6%, respectively [30], suggesting our approximate seroprevalence may be under-detecting true prevalence by an unknown margin.

## 5. Conclusions

In this study we estimated the number of swine exposed to environmental *B. pseudomallei* in three Vietnamese provinces that had been previously identified as “hotspots”. Swine are good sentinel animals for understanding environmental exposure to *B. pseudomallei*. There are numerous swine in the study area, they have high levels of contact with soil and water environments, and the relative ease of sampling in collaboration with Vietnamese veterinary and animal health services make swine well-suited to this role. As livestock are exposed to the elements, they experience higher levels of exposure during severe weather events than humans, resulting in the relatively high seroprevalence rates observed in swine. Although we sampled farmed and grazed animals from different provinces, we found grazing swine had higher exposure levels to environmental *B. pseudomallei*. Compared to a farmed animal, the amount of land a grazing animal can come into contact with is much larger and more diverse, including more numerous contaminated soil and water sources. However, if a communal water source becomes contaminated on a farm, the potential for a point-source outbreak is higher.

Domesticated food animals such as swine are in close contact with the environmental reservoir of *B. pseudomallei* and the humans that handle them. The risk infected animals pose to humans likely corresponds to the level of contact. Infected animals could understandably be sources of cutaneous, ingestional, or inhalational exposures. It is currently unclear if this occurs at an appreciable level or if any human cases of melioidosis in Vietnam are linked. At the very least, the findings presented in this work justify a continued effort to survey and characterize animal exposure to *B. pseudomallei* in the environment. In the future we will aim to understand if melioidosis is transmitted to humans through swine, characterizing a zoonotic aspect to an environmentally mediated pathogen.

### Study Limitations

This preliminary study has provided abundant information and indicated where a larger more intensive characterization of human melioidosis in Vietnam would benefit from zoonotic disease surveillance. Being a preliminary study, we were limited in reagents and availability of swine serum samples. The two sets of swine serum samples were collected from two different areas at different times of the year; meaning we are limited in the conclusions made about temporal and seasonal seroprevalence patterns between the two. The procedure for defining a seropositive threshold is somewhat arbitrary, which is a difficulty encountered by almost all serological studies. Overall seropositivity in this study was defined based on a combination of different assays, and individuals were frequently seronegative to one assay and seropositive to the other. All the grazing swine came from the same province, meaning that differences between farm and graze could be solely due to differences in prevalence between provinces. However, we note seroprevalence in grazing swine was higher across communes compared to farmed swine. In light of this, seroprevalence numbers are approximate and indicated as such. Future work will address seasonal/temporal and agricultural variability of *B. pseudomallei* exposure in sampled swine populations.

## Figures and Tables

**Table 1:** Swine sample details.

Province	District	Commune/Township	Location Code	Sample Date (M/D/YYYY)	Number of Samples (*n*)
Ha Tinh	Ky Anh	Ky Van	KV	8/8/2019	20
	Can Loc	Thuong Loc	TL	9/18/2019	32
	Nghi Xuan	Xuan Hai	XH	10/7/2019	58
	Nghi Xuan	Xuan My	XM	10/7/2019	55
	Vu Quang	Huong Minh	HM	10/7/2019	58
	Vu Quang	Vu Quang	VQ	10/7/2019	58
	Huong Son	Son Kim1	SK	10/7/2019	116
	Loc Ha	Hong Loc	HL	10/7/2019	58
	Loc Ha	Thinh Loc	TH	10/7/2019	58
	Duc Tho	Duc An	DA	10/8/2019	28
	Huong Khe	Phu Gia	PG	10/9/2019	28
	Loc Ha	Phu Luu	PL	10/9/2019	74
	Huong Son	Son Kim 1	SK	10/11/2019	45
	Vu Quang	Vu Quang	VQ	10/12/2019	55
Sub-total					743
Hoa Binh	Da Bac	Muong Chieng	MC	6/2018	50
		Giap Dat	GD	6/2018	68
		Doan Ket	DK	6/2018	60
		Tan Minh	TM	6/2018	60
		Cao Son	CS	6/2018	76
		Trung Thanh	TT	6/2018	38
Sub-total					352
Nghe An	Nam Dan	Nam Thai	NT	9/19/2019	30
Sub-total					30
Total					1125

**Table 2:** ELISA assay results summary per antigen and location.

Province (Sample Type)	Town	CPS		OPS		Hcp1		Seropositivity Rate (#Positive Samples/Total Samples; %)
		# of Tests	Au_450_ * Median (IQR ^†^)	# of Tests	Au_450_ Median (IQR)	# of Tests	Au_450_ Median (IQR)	
Ha Tinh (FARM)	KV	20/20	0.094 (0.088–0.100)	20/20	0.095 (0.087–0.101)	1/20	0.918 (0.918–0.918)	1/20; 5.0%
	TL	32/32	0.081 (0.075–0.087)	32/32	0.083 (0.071–0.101)	6/32	0.529 (0.117–0.917)	5/32; 15.6%
	XH	58/58	0.086 (0.075–0.095)	58/58	0.099 (0.084–0.114)	4/58	0.123 (0.102–0.150)	0/58; 0.0%
	XM	55/55	0.075 (0.068–0.090)	55/55	0.081 (0.074–0.109)	0/55	NP ^‡^	0/55; 0.0%
	HM	58/58	0.078 (0.07–0.090)	58/58	0.079 (0.069–0.092)	0/58	NP	0/58; 0.0%
	VQ	113/113	0.087 (0.073–0.096)	113/113	0.092 (0.080–0.104)	6/113	0.676 (0.577–0.900)	6/113; 5.31%
	SK	161/161	0.076 (0.070–0.086)	161/161	0.079 (0.069–0.095)	1/161	0.217 (0.217–0.217)	1/161; 0.62%
	HL	58/58	0.073 (0.067–0.083)	58/58	0.094 (0.081–0.109)	0/58	NP	0/58; 0.0%
	TH	58/58	0.071 (0.065–0.086)	58/58	0.094 (0.079–0.105)	0/58	NP	0/58; 0.0%
	DA	28/28	0.089 (0.079–0.093)	28/28	0.086 (0.077–0.097)	0/28	NP	0/28; 0.0%
	PG	28/28	0.086 (0.080–0.094)	28/28	0.088 (0.085–0.103)	0/38	NP	0/38; 0.0%
	PL	74/74	0.084 (0.076–0.095)	74/74	0.117 (0.099–0.248)	19/74	0.201 (0.177–0.382)	18/74; 24.3%
Sub-total		743/743	0.080 (0.056–0.091)	743/743	0.089 (0.076–0.105)	37/743	0.217 (0.168–0.552)	31/743; 4.17%
Hoa Binh (GRAZE)	MG	50/50	0.104 (0.09–0.104)	50/50	0.116 (0.097–0.134)	4/50	0.272 (0.254–0.758)	4/50; 8.0%
	GD	68/68	0.098 (0.086–0.098)	68/68	0.115 (0.099–0.141)	7/68	0.232 (0.162–0.324)	6/68; 8.8%
	DK	60/60	0.099 (0.092–0.099)	60/60	0.100 (0.089–0.147)	4/60	0.239 (0.178–1.316)	4/60; 6.7%
	TM	60/60	0.104 (0.085–0.104)	60/60	0.111 (0.093–0.146)	6/60	0.236 (0.194–0.303)	6/60; 10.0%
	CS	30/76	0.095 (0.086–0.095)	30/76	0.096 (0.087–0.115)	48/76	0.240 (0.185–0.348)	13/76; 17.1%
	TT	15/38	0.082 (0.073–0.082)	15/38	0.088 (0.077–0.126)	24/38	0.184 (0.138–0.246)	7/38; 18.4%
Sub-total		283/352	0.098 (0.061–0.11)	283/352	0.110 (0.091–0.140)	93/352	0.232 (0.174–0.295)	40/352; 11.36%
Nghe An (FARM)	NT	30/30	0.071 (0.068–0.077)	30/30	0.073 (0.068–0.080)	3/30	0.213 (0.192–0.215)	0/30; 0.00%
Sub-total		30/30	0.071 (0.068–0.077)	30/30	0.073 (0.068–0.080)	3/30	0.213 (0.192–0.215)	0/30; 0.00%
Total		1056/1125	0.084 (0.056–0.097)	1056/1125	0.093 (0.078–0.114)	133/1125	0.232 (0.177–0.346)	71/1125; 6.31%

* Au450 = absorbance at 450 nm; ^†^ IQR = interquartile range; ^‡^ NP = not performed.
